# Dramatic response of metaplastic breast cancer to chemo-immunotherapy

**DOI:** 10.1038/s41523-017-0011-0

**Published:** 2017-03-29

**Authors:** Sylvia Adams

**Affiliations:** New York University School of Medicine, Perlmutter Cancer Center, New York, NY USA

## Abstract

Frequent overexpression of programmed death-ligand 1 has recently been demonstrated in metaplastic breast cancer, which is a rare breast cancer subtype with limited treatment options. This report describes the clinical course of a patient with metastatic metaplastic breast cancer who had a remarkable response to anti-programmed death-1 therapy with pembrolizumab in combination with nab-paclitaxel. Tissue correlates are presented including tumor-infiltrating lymphocytes and high-programmed death-ligand 1 expression in the tumor.

Metaplastic breast cancers (MPBC) are rare and very aggressive tumors comprising ~1% of all breast cancers. These tumors are typically composed of adenocarcinoma and metaplastic (squamous, chondroid, spindle, rhabdoid, or osseous) elements.^1^ While the biomarker profile is commonly ‘‘triple’’ negative (ER/PR and HER2 negative), the prognosis of MPBC is even worse than non-MPBC triple-negative breast cancers (TNBC) with a poor response to systemic therapy, and a median survival of 8 months after the development of metastases.^2–4^

Given the limited treatment options and poor prognosis of metastatic MPBC novel treatments are urgently needed. Coinciding with a recent publication of frequent overexpression of programmed death (PD)-ligand 1 (PD-L1) in primary MPBC,^5^ we observed a remarkable response of a bulky and widely metastatic MPBC in an ongoing clinical trial of chemotherapy with pembrolizumab (anti PD-1 antibody, clinical trials.gov NCT02752685).

The patient is a 53-year-old woman (BRCA negative) who originally discovered a mass in her right breast in June 2015. Based on an excisional biopsy a metaplastic carcinoma was diagnosed. A positron emission tomography computed tomographic (PET CT) scan was negative for distant metastases. Neoadjuvant treatment was not recommended as MPBC was considered non-responsive; therefore, the patient proceeded with a right modified radical mastectomy. Pathology confirmed a high-grade triple-negative spindle cell metaplastic carcinoma of the breast, stage pT3N1a. After only three cycles of adjuvant adriamycin and cyclophosphamide the same histologic tumor recurred in the right chest wall and lungs. She was treated with liposomal doxorubicin, bevacizumab, and everolimus from December 2015–February 2016 and a Phase I study of gemcitabine, carboplatin, and interleukin-10 from March 2016–May 2016, but experienced disease progression. She presented to our center for participation in an immunotherapy trial, consented and started treatment in July 2016 on a Phase 2 clinical trial of nab-paclitaxel with pembrolizumab. This study includes serial biopsies to assess changes in the tumor microenvironment from baseline to chemotherapy alone (cycle 1) and then the chemoimmunotherapy combination (cycle 2 and subsequent cycles). The study treatment consisted of nab-paclitaxel (d1 and 8 intravenously at 100 mg/m^2^) and pembrolizumab (d1 intravenously at 200 mg, starting with cycle two); cycles were repeated every 3 weeks.

On baseline physical exam there was a large ulcerating malodorous chest wall mass (Fig. 1a). The baseline PET CT scan revealed an intensely Fludeoxyglucose (FDG) avid large infiltrative multilobulated heterogeneous soft tissue mass within the right anterior chest wall extending into the right axilla and associated osseous destruction of the sternum, several ribs as well as invasion into the pleural surface (Fig. 2a). Furthermore, it showed multiple FDG avid regional nodal metastases, extensive bilateral lung parenchymal (which were biopsy proven), pleural and osseous metastases (Fig. 2c). After the first treatment cycle with nab-paclitaxel alone there was significant tumor progression on visual examination (Fig. 1b), worsening of the patient’s performance status and new onset malignant hypercalcemia. She received intravenous fluids, furosemide, and pamidronate with normalization of calcium levels and went on to receive the second cycle, now including the PD-1 inhibitor pembrolizumab along with nab-paclitaxel. Following the first dose of pembrolizumab, the patient’s performance status improved dramatically and the malignant wound had significantly diminished with appearance of granulation tissue (Fig. 1c). Adjacent to the tumor bed a soft protruding mass expanded, followed by drainage of necrotic material weeks later and complete disappearance (Fig. 1e). After the second dose of pembrolizumab the wound started to epithelialize (Fig. 1d) and healed 2 months later (Fig. 1e). The 9-week PET CT scan confirmed a marked anatomic and metabolic response in chest wall, nodal, osseous, and pulmonary metastases (Fig. 2b, d). In fact the tumor shrinkage in pleura and lung with resultant parenchymal retraction resulted in interval enlargement of multiple cavitary foci associated with metastases, and the development of small bilateral pneumothoraces, which ultimately required chest tubes and pleurodesis. She continues to receive study treatment and is doing well with continuing tumor response at 6 months.

**Fig. 1:**
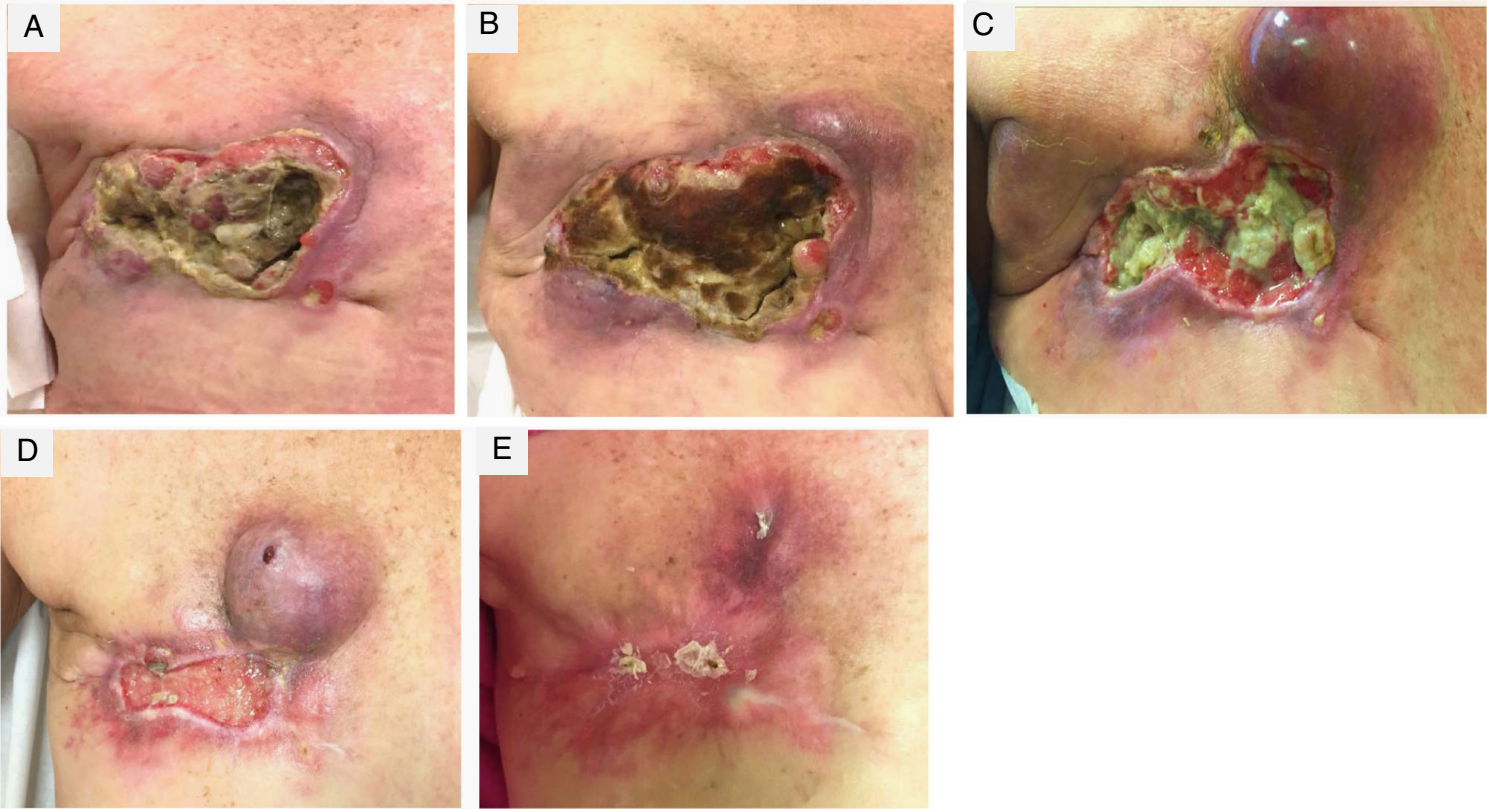
Clinical response of a large chest wall recurrence of metaplastic breast cancer to Pembrolizumab/nab-paclitaxel after progression on single agent nab-paclitaxel. Photograph at **a** baseline, **b** after 1st treatment cycle (nab-paclitaxel alone), **c** after two cycles (nab-paclitaxel and pembrolizumab), **d** after three cycles and **e** after six cycles

**Fig. 2:**
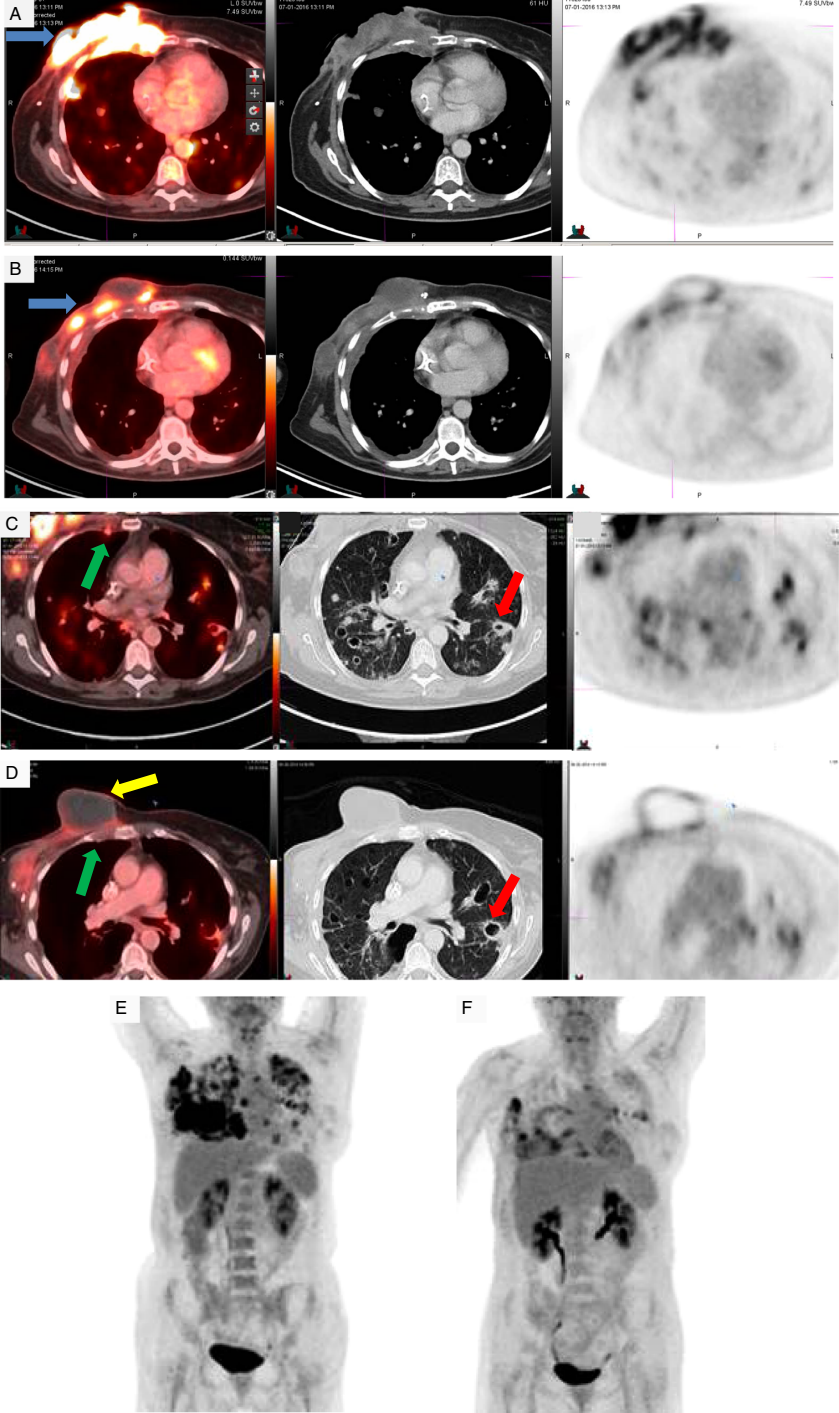
Radiographic response of recurrent metaplastic breast cancer with a bulky chest wall mass and pulmonary metastases to pembrolizumab/nab-paclitaxel. PET CT images **a** at baseline depicting the large and destructive chest wall mass (SUV 11.0, 10.1 × 3.6 cm, *blue arrow*), invading ribs, sternum, and pleura, **b** at 9 weeks (s/p three cycles) demonstrating a significant metabolic and anatomic response on the chest wall mass (SUV 5.6, 6.5 × 4.7 cm, *blue arrow*), **c** at baseline depicting bilateral lung parenchymal (*red arrow*) and nodal metastases (*green arrow*), **d** at 9 weeks demonstrating a decreased number, size, and metabolic activity of pulmonary metastases and worsening cavitation (*red arrow*) as sign of favorable response to therapy. Additionally, the protruding mass (seen on the clinical photographs) is shown with necrotic debris. Complete response to the previously identified intensely FDG avid necrotic right internal mammary chain lymph node (*green arrow*). **e** Coronal image at baseline depicting the bulky right chest wall recurrence, extensive bilateral lung parenchymal and pleural metastases as well as nodal metastases. **f** Coronal image at 9 weeks demonstrating marked interval improvement in the right chest wall mass consistent with a partial treatment response, as well as interval decrease of multiple FDG avid nodal, pulmonary, and pleural metastases

PD-L1 staining was performed by Qualtek (Newtown, PA) on formalin-fixed paraffin-embedded tumor sections obtained at study baseline utilizing immunohistochemistry (IHC, 22C3 antibody, Merck). Results showed 100% of tumor cells staining for PD-L1 and an increase in tumor-infiltrating lymphocytes (TILs) after pembrolizumab treatment was observed (Fig. 3).

**Fig. 3:**
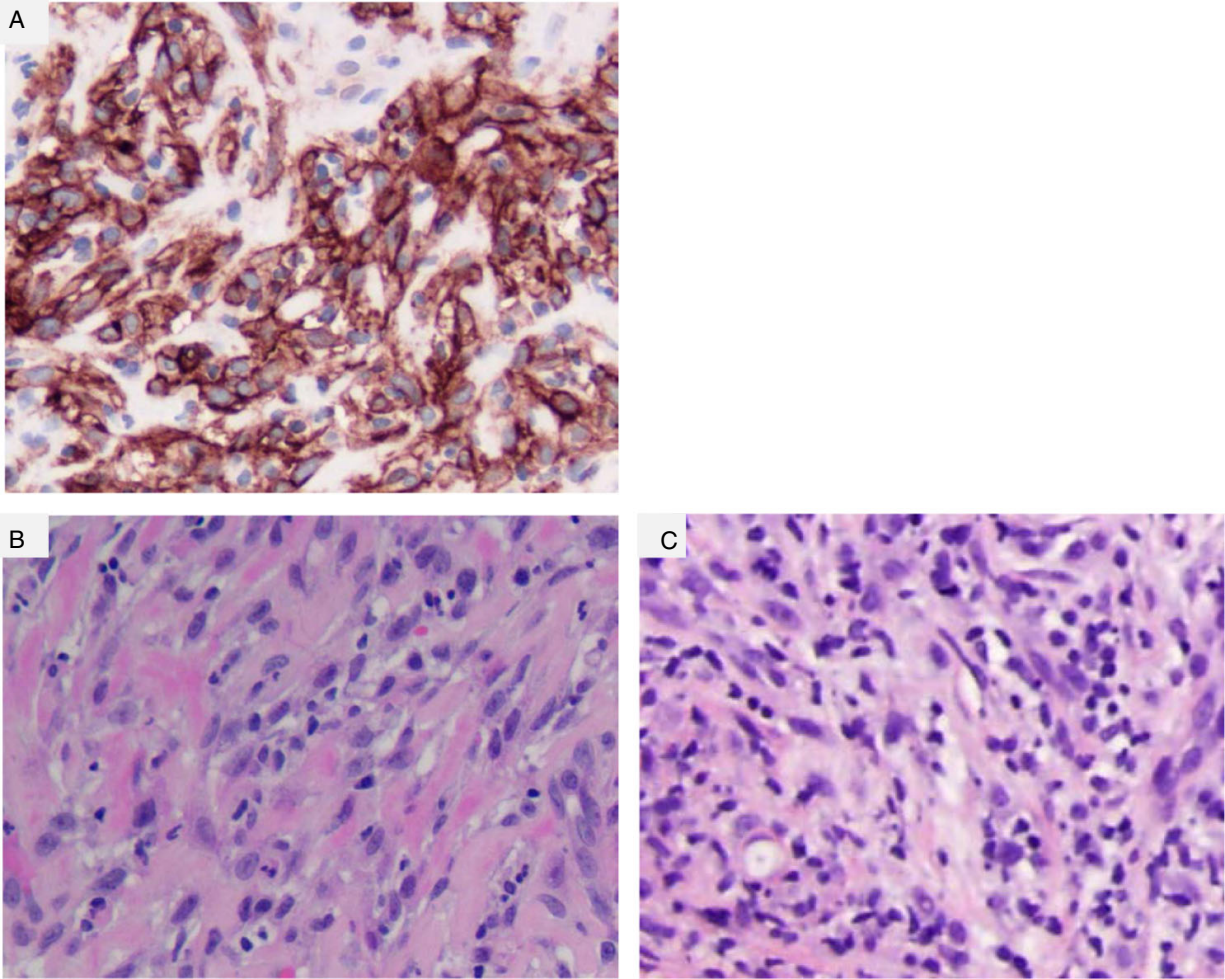
Histopathologic features of the MPBC obtained by punch biopsy from peripheral chest wall lesion (inferolateral margin). **a** Baseline PD-L1 IHC stain at 20×. One-hundred percent of tumor cells stain positive for PD-L1 (95% at 3 + intensity, 5% at 2 + intensity), **b** Tumor H and E stain at 40× at baseline and **c** after treatment with pembrolizumab (biopsy cycle 3, day 1) with increase in TIL from baseline (10 to 30%)

These correlative tissue results are in concordance with the study by Joneja *et al*.,^5^ which showed that MPBC commonly overexpress PD-L1, which is in stark contrast to other breast cancer subtypes including TNBC. In their study of MPBC they found that tumor cell PD-L1 expression, defined as =/> 5% cells, was seen in 33/72 (46%) cases. In addition, TILs are frequently observed in MPBC, and strongly PD-1-positive TILs have been described in half of the PD-L1-negative MPBC,^5^ consistent with an immunogenic cancer phenotype, which may result from the higher mutation frequency and gene copy number variance in MPBC compared to other types of breast cancer.^6^

Among the mechanisms by which tumor cells can regulate PD-L1 expression are oncogenic alterations such as the PTEN/PI3K and the Ras–MAPK pathways, which have been shown to contribute to immune evasion in breast cancer.^7, 8^ MPBC are enriched for PIK3CA mutations when compared with TNBC,^5^ and the patient presented here also carries a PIK3CA mutation (H1047R), which may account for tumoral PD-L1 expression. However, in a recent series of MPBC, no statistically significant correlation was identified between the number of molecular alterations or types of mutations and PD-L1 expression,^5^ therefore, other causes of tumoral PD-L1 expression such as genomic amplification of 9p24.1 described in TNBCs^9^ could contribute. Interestingly, PD-L1 expression can also be upregulated by the induction of epithelial to mesenchymal transition,^10^ which is a typical feature of MPBC.^11–13^

This patient had a rapid eradication of a large tumor mass and visceral metastases (Fig. 2e, f) with two doses of pembrolizumab given in combination with nab-paclitaxel. While the contribution of chemotherapy to this response cannot be ruled out, the patient had clinically significant progression on nab-paclitaxel administered as single agent during cycle one, which suggests a powerful effect of immunotherapy in treatment-refractory MPBC. We wish to bring this to the attention of our colleagues for two reasons. First, as an example of the exciting potential of immunotherapy in breast cancer, including a rare breast cancer subtype, which was recently shown to commonly overexpress PD-L1 and to encourage patient referral into trials of immune checkpoint inhibitors. Second, dramatic and rapid tumor shrinkage can lead to complications in the underlying non-cancerous tissue such as the pneumothoraces described here, therefore, careful monitoring of patients is required.
